# The effect of liquid platelet-rich fibrin on oral cells and tissue engineered oral mucosa models in vitro

**DOI:** 10.1038/s41598-025-11868-0

**Published:** 2025-07-18

**Authors:** Krit Rattanawonsakul, George Seleiro, Victoria Workman, Frederik Claeyssens, Robert Bolt, Peeratchai Seemaung, Vanessa Hearnden

**Affiliations:** 1 School of Chemical, Materials and Biological Engineering, The University of Sheffield, Sheffield, S3 7HQ UK; 2 Department of Pharmacology, Faculty of Dentistry, Mahidol University, Bangkok, Thailand; 3 School of Clinical Dentistry, The University of Sheffield, Sheffield, S10 2TA UK; 4 INSIGNEO Institute, The University of Sheffield, Sheffield, UK

**Keywords:** Platelet rich fibrin, Wound healing, Tissue engineered model, Fibroblast, Keratinocyte, Dentistry, Tissues

## Abstract

Liquid formulations of platelet-rich fibrin (Liquid-PRF) have been shown to promote oral soft tissue healing for some clinical applications, however, the efficacy of liquid-PRF as a standalone treatment remains uncertain. The aim of the present study was to investigate the effects of liquid-PRF on oral cells in vitro using two-dimensional cell culture and three-dimensional tissue-engineered oral mucosa models. Media was conditioned with liquid-PRF prepared from blood samples and applied to oral fibroblasts, keratinocytes and tissue-engineered oral mucosa models. Metabolic activity, migration, proliferation and epithelial morphology were assessed. Liquid-PRF was shown to be biocompatible, with no cytotoxic effects observed on oral mucosa cells or 3D oral mucosa models. Cytokine analysis confirmed the presence of key growth factors, including PDGF-BB, TGF-β1, and EGF. Liquid-PRF increased oral fibroblast proliferation and promoted keratinocyte migration in 2D cultures. In tissue-engineered oral mucosa models, liquid-PRF showed no significant improvement in metabolic activity, epithelium thickness, morphology or proliferative capacity. The results suggest that growth factors in liquid-PRF were able to stimulate the proliferation and migration of oral mucosa cells in 2D culture, however these effects could not be demonstrated in 3D oral mucosa models. Factors secreted from liquid PRF were able to support the growth of cells and the development and maintenance of a healthy epithelium. Despite improvements in keratinocyte migration and fibroblast proliferation the results from 3D models indicate that factors secreted from liquid-PRF alone may not be sufficient to stimulate oral soft tissue repair.

## Introduction

Platelet concentrates have emerged as powerful tools in regenerative medicine, offering significant therapeutic potential across diverse applications, including skin rejuvenation, musculoskeletal repair, and wound healing within the oral and maxillofacial region^1^. Platelet-rich plasma (PRP), was introduced in the 1990s to deliver supraphysiological concentrations of platelets and growth factors; however, the inclusion of anticoagulants led to limitations in its use^2–4^.

Platelet-rich fibrin (PRF) is a gel-like formulation produced through one-step centrifugation, that eliminates the need for additional chemical agents^5,6^. The three-dimensional (3D) network of PRF entraps growth factors and inflammatory cells^7^ providing a slow continuous release of growth factors over 10 days and offering a more prolonged effect than PRP^8,9^. PRF is biocompatible and non-immunogenic as it can be derived from an autologous source^5^. Recent advancements using low-speed and shorter centrifugation times have led to the development of liquid-PRF or injectable-PRF^5^. Liquid-PRF is versatile and has been combined with materials such as bone grafts or biological scaffolds, broadening its clinical applicability^10–12^.

Liquid-PRF has demonstrated potential in promoting soft tissue regeneration for some dental applications. A randomised controlled trial demonstrated the benefits of liquid-PRF in wound healing and re-epithelialization after gingivectomy and gingivoplasty in patients with gingival overgrowth^13^. Combining liquid-PRF with connective tissue grafts and coronally advanced flaps yielded better outcomes for gingival recession than treatments without liquid-PRF^14^. However, the effectiveness of liquid-PRF as a standalone therapy for oral mucosa regeneration is still debated.

Oral mucosa wound healing is a complex process involving a series of highly coordinated events, involving fibroblasts, keratinocytes, endothelial cells, and inflammatory cells^15,16^. Key mediators of these events are derived from platelets, which release growth factors during the clotting process, leukocytes, which contribute to inflammation and tissue remodelling, and surrounding mucosal tissues that provide additional signals and matrix structures for regeneration and repair^17,18^. Liquid-PRF, has been investigated for its potential to provide a slow release of regenerative factors for soft tissue oral wound healing^5,19–21^.

A deeper exploration of the biological roles of liquid-PRF is necessary to unlock its potential in clinical and research settings. An increased understanding of how liquid-PRF and factors secreted from it affect cell and tissue behaviour in the oral mucosa will support the development and optimisation of platelet concentrate treatments for wound healing in the oral and maxillofacial region. A previous study by Miron et al., demonstrated that gingival fibroblasts treated with liquid-PRF in vitro had increased expression of genes related to wound healing and regeneration, including platelet-derived growth factor (PDGF), transforming growth factor-β (TGF-β), and collagen^5^. However, we must also consider the role of keratinocytes and the behaviour of cells in three-dimensions^22,23^. Tissue-engineered oral mucosa (TEOM) models, which incorporate fibroblasts and keratinocytes with a lamina propria, offer a more physiologically relevant model for these investigations^24^.

The aim of the present study was to investigate how factors secreted from liquid-PRF affected oral mucosa cell and tissue function, using 2D cell culture and 3D TEOM models.

## Materials and methods

### Ethics approval and consent to participate

The protocol for blood collection and liquid-PRF processing was ethically approved by the University of Sheffield Research Ethics Committee (REC) (Reference number: 034492) and the National Health Service (NHS) REC (Reference number: 22/NW/0034). The protocols for the collection, processing, and use of human tissues (including de-cellularised dermis (DED)) and oral cells in this study were approved by the University of Sheffield Research Ethics Committee (Reference number: 003463) and the NHS Ethics Committee (Reference numbers: 15/YH/0177 and 21/NE/0115). Written informed consent was obtained from all volunteers prior to any procedures being performed. All procedures were performed in accordance with the Declaration of Helsinki.

### Blood collection and liquid-PRF preparation

**Fig. 1:**
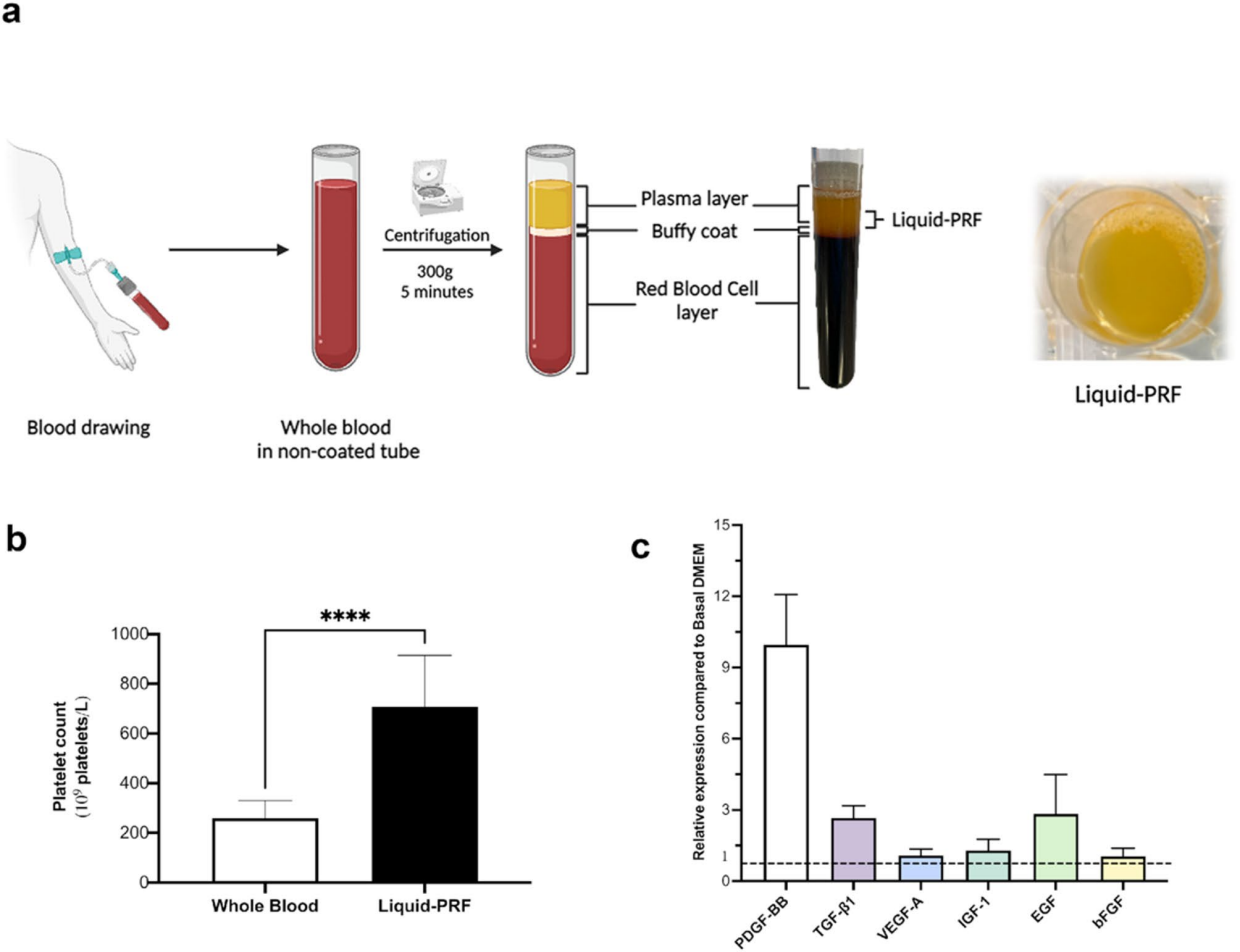
Liquid-PRF preparation process and its biological contents. Blood samples were collected from volunteers into tubes without anticoagulants and subsequently centrifuged at 300 g for 5 min. Liquid-PRF was collected from the interface between the yellow serum and the buffy coat layers (**a**). Platelet count of whole blood and liquid-PRF (Mean ± standard deviation (S.D.) *N* = 9). Statistical analysis was determined using an unpaired t-test (**** *p* < 0.0001) (**b**). The relative expression of growth factors presented in liquid-PRF compared to basal DMEM (Mean ± S.D. *N* = 3) (**c**). Abbreviations; bFGF, basic fibroblast growth factor; EGF, epidermal growth factor; IGF-1, insulin-growth factor-1; PDGF-BB, platelet-derived growth factor; TGF-β1, transforming growth factor-beta 1; VEGF-A, vascular endothelial growth factor-A. Partially created in BioRender. Hearnden, V. (2025) https://BioRender.com/w3gtc5l.

Peripheral blood samples were collected from nine healthy volunteers with written informed consent. Individuals who were under 18 years old, unable or unwilling to provide informed consent, those working directly with the samples, and those with known blood-borne infections were excluded from this study. Blood samples were drawn into 9-mL non-coated tubes (Greiner Bio-One, UK) and centrifuged at 300 g (1,310 rpm) for 5 min using a horizontal-swing rotor centrifuge (320R Universal, Hettich, UK)^20^. Liquid-PRF was harvested from the interface above the yellow-red junction using an 18G blunt-fill needle (BD Biosciences, UK) and prepared as shown in (Fig. 1a)^20^. Platelet counts were performed on whole blood collected in K2-EDTA-coated tubes (Greiner Bio-One, UK) to prevent coagulation.

### Conditioned medium preparation

Liquid-PRF (1 mL) was incubated at 37^o^C, in a humidified atmosphere containing 5% CO_2_ for 1 h before Dulbecco’s Modified Eagle Medium (DMEM, Sigma-Aldrich, UK) with no supplements was added at a ratio of 1:5 (Liquid-PRF: basal DMEM) and conditioned medium was collected after 72 h.

### Cytokine array

The RayBio^®^ C-Series human cytokine antibody array C1000 kit (RayBioTech, USA) was used according to the manufacturer’s instructions, to detect growth factors in conditioned medium derived from liquid-PRF. The array membranes were blocked with blocking buffer for 30 min at room temperature, then incubated overnight at 4^o^C with the conditioned medium derived from liquid-PRF. The next day, membranes were washed and were incubated with a biotinylated antibody cocktail overnight at 4^o^C, followed by incubation with horseradish peroxidase (HRP)-Streptavidin solution for 24 h. Chemiluminescence signals were detected using a C-DiGit scanner (LI-COR Biosciences, UK) and densitometry was performed using ImageJ software (National Institute of Health). The relative expression of each growth factor in the conditioned medium were determined by normalising to the positive control on the corresponding membrane and to the control membrane incubated with basal medium in the absence of liquid-PRF.

### Cell culture

An immortalised oral keratinocyte cell line expressing telomerase reverse transcriptase (FNB6/TERT) and primary human normal oral fibroblasts (NOFs) were used in this study. FNB6/TERT, derived from buccal mucosa biopsies^22^ were kindly provided by the School of Clinical Dentistry, The University of Sheffield, and The Beatson Institute for Cancer Research, Scotland. FNB6/TERT cells were cultured in complete Green’s medium comprising of a 3:1 mixture of DMEM and Ham’s Nutrient Mixture F12 (Sigma-Aldrich, UK) and supplemented with 10% foetal bovine serum (FBS, Biosera, UK), 0.01 mg/ml L-glutamine, 100 µg/ml Penicillin/Streptomycin, 0.625 µg/ml Fungizone, 0.025 µg/ml adenine, 1.36 ng/ml/5 µg/ml of 3,3,5-Tri-iodothyronine/Apo-Transferrin (T/T), 5 ng/ml epidermal growth factor (EGF), 5 µg/ml insulin, 4 µg/ml hydrocortisone, and 8.47 ng/ml cholera toxin (all from Sigma-Aldrich, UK).

NOFs were isolated from buccal tissues as described previously^23^. The epithelium was removed and the remaining lamina propria was minced and digested in 0.05% (w/v) Collagenase A (Merck, UK). NOFs were cultured in DMEM supplemented with 10% FBS, 1% Penicillin/Streptomycin and 1% L-Glutamine.

For the experiments, oral fibroblasts (between passages 6 and 10) were treated in basal DMEM without supplements (PRF 0%), basal DMEM supplemented with 10%, 20%, or 50% liquid-PRF-conditioned medium, or basal DMEM containing 10% FBS. FNB6/TERT cells (between passages 15 and 24) and TEOM were treated in either Green’s medium without FBS and EGF, referred as basal Green’s medium (PRF 0%), basal Green’s medium supplemented with 10%, 20%, or 50% liquid-PRF-conditioned medium, or complete Green’s medium (FBS 10%).

### Metabolic activity assay

NOFs and FNB6/TERT cells were seeded at densities of 10,000 cells/cm^2^ and 16,700 cells/cm^2^, respectively and allowed to adhere for 24 h. Cells were incubated with conditioned medium for 72 h, and the metabolic activity was evaluated at 24 and 72 h using the 3-(4,5-dimethylthiazol-2-yl)−2,5-diphenyltetrazolium bromide (MTT) (Sigma-Aldrich, UK) assay. At each time point, cells were incubated with 0.5 mg/mL MTT solution for 90 min. Acidified isopropanol was then added to solubilise the formazan crystals and absorbance was read at 540 nm with a reference reading at 630 nm.

### Proliferation assay

Cell proliferation was measured by incubating NOFs and FNB6/TERT with 1 µM CellTrace™ Far Red carboxy-fluorescein diacetate N-succinimidyl diester (CFSE) (Thermo Fisher Scientific, UK) for 72 h. Non-proliferative controls were generated by treating cells with mitomycin C (2 mg/mL for NOFs, 0.5 mg/mL for FNB6/TERT) for 4 h. Mean fluorescence intensity (MFI) was measured using an LSRII flow cytometer (BD Bioscience, UK) (488 nm excitation 530 nm emission). The proliferation index was calculated by comparing the MFI of each experimental condition to the value of the mitomycin C-treated control.

### Migration assay

The effect of liquid-PRF on the migration of oral mucosa cells was measured using the Oris™ migration assay (Platypus Technologies, USA). NOFs (54,000 cells/cm^2^) and FNB6/TERT at (132,000 cells/cm^2^) were seeded into 96-well plates and a defined cell free region was created with the Oris™ stoppers. After 24 h, mitomycin C was added to inhibit cell proliferation. Cells were incubated with conditioned medium and imaged every 24 h up to 72 h for NOFs, and every 8 h up to 24 h for FNB6/TERT using an inverted light microscope (Motic AE2000) with a digital camera (Moticam 2). The cell-free region was measured using ImageJ software and expressed as a percentage of the initial area.

### Tissue-engineered oral mucosa construction and treatment with liquid-PRF

TEOM was cultured as described previously^25^. In brief, NOFs (250,000 cells in 0.25 mL) and FNB6/TERT (1 × 10^6^ cells in 0.25 mL) were seeded simultaneously within a custom-made stainless-steel chamfered ring placed on a DED. DED was generated from waste skin collected from routine surgical procedures, collected with written informed consent. Skin was grafted to approximately 1 mm thickness and incubated in 1 M sodium chloride (NaCl) solution at 37 °C for 24 h to enable the epithelium and cellular components to be removed. Following cell seeding TEOM was submerged in Green’s medium for 72 h and incubated at 37^o^C in a humidified atmosphere with 5% CO_2_. TEOM was transferred to 12-mm transwell inserts with 0.4 μm pore diameter (THINCERT, Greiner Bio-One, UK) and placed in 12-deep well plates (Greiner Bio-One, UK) to establish an air-liquid interface (ALI). TEOM was treated with the conditioned medium when raised to ALI and cultured for 10 days.

### Metabolic activity assay for TEOM constructs

TEOM was incubated with resazurin solution (0.1mM) (STEMCELL™ Technologies, UK) at 37^o^C for 4 h to measure metabolic activity and fluorescence was measured using an FLx800 fluorescence microplate reader (BioTek, UK) (562 nm excitation/630 nm emission).

### Histology and haematoxylin and eosin staining

Formalin-fixed TEOM samples were processed through standard histological procedures and sectioned into 5 μm thick slices. Slides were stained with haematoxylin and eosin (H&E) and images of the TEOM were captured using an Olympus CX43 inverted light microscope, equipped with a Euromex VC.3036 HD-Ultra camera. Image backgrounds were removed using Adobe Photoshop 2023, and brightness, contrast, and scale bars adjusted uniformly using ImageJ software.

### Epithelium thickness measurement

Epithelial regions were manually highlighted using the polygon tool and average epithelium thickness was quantified using the Simple Interactive Object Extraction (SIOX) plugin in ImageJ/Fiji software^26^.

### Immunohistochemistry staining of Ki-67

Ki-67 staining was performed to identify proliferating cells. TEOM sections were incubated with hydrogen peroxide solution for 30 min and antigen retrieval was performed with sodium citrate buffer (pH 6.0) for 2 h. After washing with phosphate-buffered saline (PBS) containing 0.05% Tween-20 (Sigma-Aldrich, UK), sections were blocked with protein block solution for 30 min, and incubated overnight at 4 °C with a ready-to-use mouse Ki-67 primary antibody (MIB-1 clone, DAKO Omnis, UK). Sections were then incubated with biotinylated goat anti-polyvalent antibody for 1 h, followed by Streptavidin-peroxidase for 30 min. Sections were developed with DAB solution (Abcam, UK) for 5 min and counterstained with haematoxylin solution. Slides were imaged using an Olympus CX43 microscope with a Euromex VC.3036 HD-Ultra camera. Ki-67-positive cells were quantified by a blinded observer. Cells with positive Ki-67 nuclear staining were expressed as a percentage of the total number of nuclei in each image.

### Statistical analyses

Data are presented as the mean ± standard deviation (SD). Data analysis was performed using GraphPad Prism 9 software (Massachusetts, USA). The Shapiro-Wilk test was applied to test the normality of the data. Statistical analysis used for each figure are detailed in the legends. Data was considered statistically significant when *p* < 0.05.

## Results

### Characterisation of liquid-PRF

Liquid-PRF was successfully collected from donor human blood using the horizontal-swing centrifuge method. Liquid-PRF had a yellowish appearance and approximately 1 mL of liquid-PRF was obtained per 9 mL blood sample (Fig. 1a). Liquid-PRF exhibited a significantly higher platelet concentration (approximately 680 × 10⁹ platelets/L) compared to whole blood (approximately 245 × 10⁹ platelets/L) (Fig. 1b).

The presence of bioactive components released from liquid-PRF into the conditioned medium was measured using a cytokine array. Our data revealed detectable levels of PDGF-BB, TGF-β1, and EGF, which were 10, 2.7, and 2.8 times higher compared to basal DMEM (without FBS), respectively (Fig. 1c). Vascular endothelial growth factor-A (VEGF-A), insulin growth factor-1 (IGF-1), and basic fibroblast growth factor (bFGF) were detected at levels comparable to those observed in basal DMEM.

### Liquid-PRF increased the metabolic activity and proliferation of oral mucosa cells in 2D culture

**Fig. 2:**
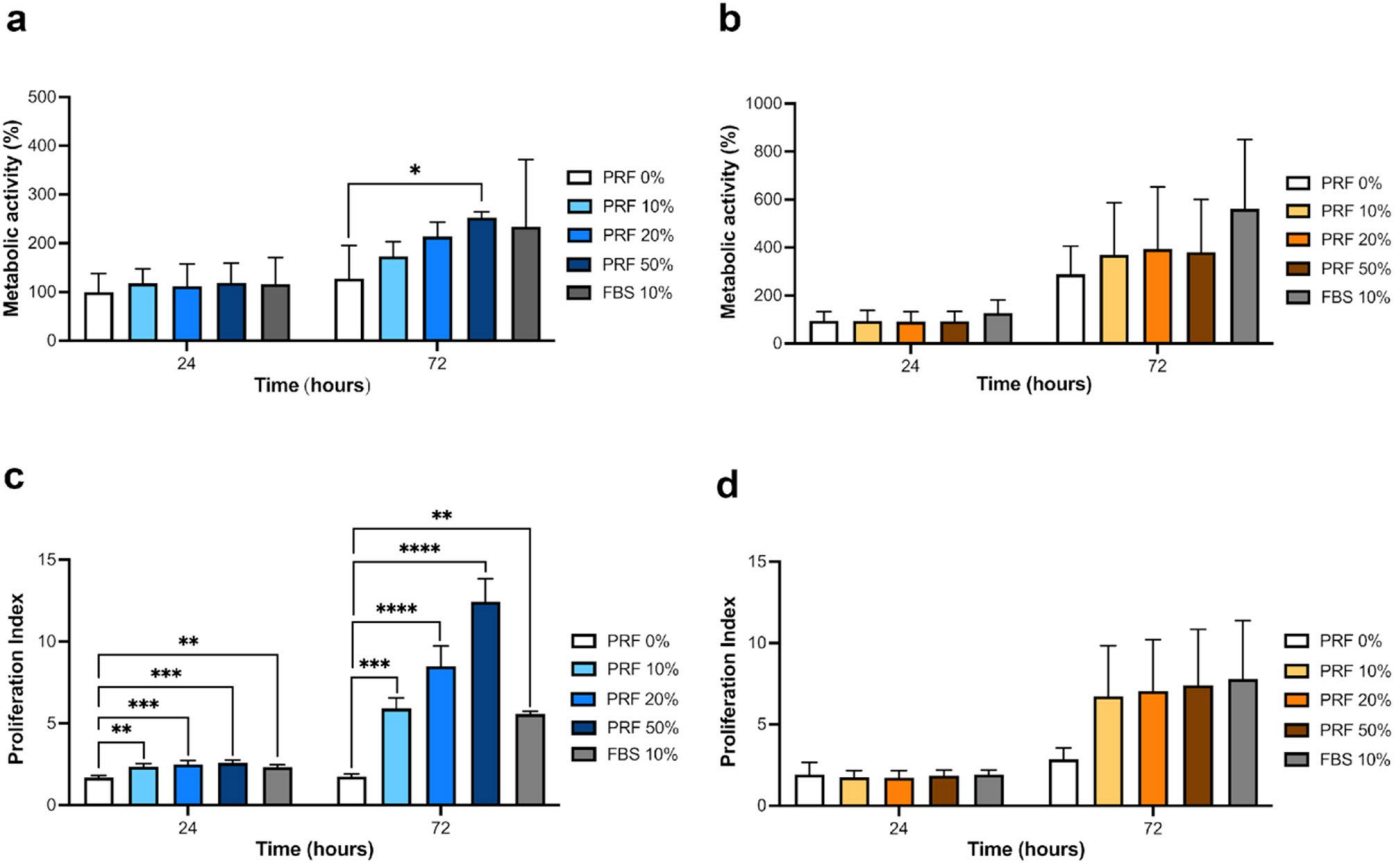
Cell metabolic activity and proliferation. The metabolic activity of NOFs (*N* = 4 or 3, *n* = 3) (**a**) and FNB6/TERT (*N* = 5 or 4, *n* = 3) (**b**) and proliferation index of NOFs (*N* = 3, *n* = 3) (**c**) and FNB6/TERT (*N* = 3, *n* = 3) (**d**) in response to conditioned medium derived from liquid-PRF over 72 h. Metabolic activity is presented relative to the control (PRF 0%). Mean ± standard deviation (S.D). Statistical significance was determined using an ordinary one-way ANOVA followed by Dunnett’s multiple comparison against the control (PRF 0%) at each time point (* *p* < 0.05, ** *p* < 0.01, *** *p* < 0.001, and **** *p* < 0.0001). Abbreviations; FBS, foetal bovine serum; PRF, platelet-rich fibrin.

The metabolic activity of NOFs after treatment with different concentrations of liquid-PRF conditioned medium over 72 h was determined using an MTT assay (Fig. 2a). There was no significant change in the NOF metabolic activity after 24-hours of culture. After 72 h, the metabolic activity of NOFs increased in a dose-dependent fashion. NOFs incubated with the 50% conditioned medium had significantly higher metabolic activity compared to the control (PRF 0%). FNB6/TERT keratinocytes had a greater increase in metabolic activity over the experimental period (200%) compared to the NOFs (25%), indicating a higher metabolic rate compared to NOFs (Fig. 2b). At 24 h, no concentration of conditioned medium affected FNB6/TERT metabolic activity. A slight increase in metabolic activity was seen after treating cells with the liquid-PRF conditioned medium for 72 h; however, no statistical significance was observed.

The proliferative index of NOFs treated with liquid-PRF-conditioned medium and FBS 10% was significantly increased compared to the control (PRF 0%) after 24 h and 72 h, with a dose dependent response observed at the later time point (Fig. 2c).

There was no significant change in FNB6/TERT proliferation in the presence of liquid-PRF conditioned medium after 24 hours or 72 hours of treatment despite the increase in proliferation index seen after 72 h (Fig. 2 d).

### Liquid-PRF increased the migration of keratinocytes but not fibroblasts in 2D culture

**Fig. 3:**
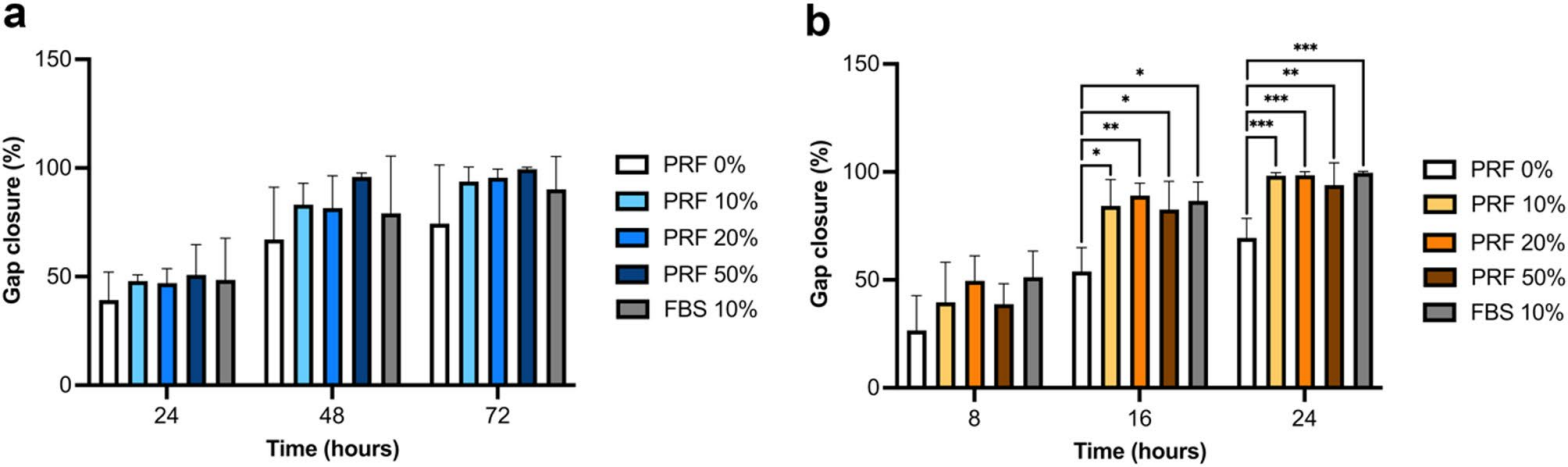
Cell migration. Percentage of in vitro gap closure of NOFs (**a**) and FNB6/TERT (**b**) in response to conditioned medium derived from liquid-PRF (*N* = 3, *n* = 3). Mean ± standard deviation (S.D). Statistical significance was determined using an ordinary one-way ANOVA followed by Dunnett’s multiple comparison against the control (PRF 0%) at each time point (* *p* < 0.05, ** *p* < 0.01, and *** *p* < 0.001). Abbreviations; FBS, foetal bovine serum; PRF, platelet-rich fibrin.

Cell migration plays a vital role in the healing process as cells migrate across the wound bed to close the wound. There was no significant difference in the degree of gap closure in NOF treated with liquid-PRF conditioned media compared to the negative control (PRF 0%), and the degree of gap closure was comparable to the positive control (FBS 10%), with over 97% closure (Fig. 3a).

The degree of gap closure with FNB6/TERT treated with liquid-PRF conditioned media was significantly higher than the negative control (PRF 0%) and comparable to the positive control (FBS 10%) after 16 and 24 h (Fig. 3b).

### Liquid-PRF produced no significant effect on the epithelial thickness or metabolic activity of TEOM

**Fig. 4:**
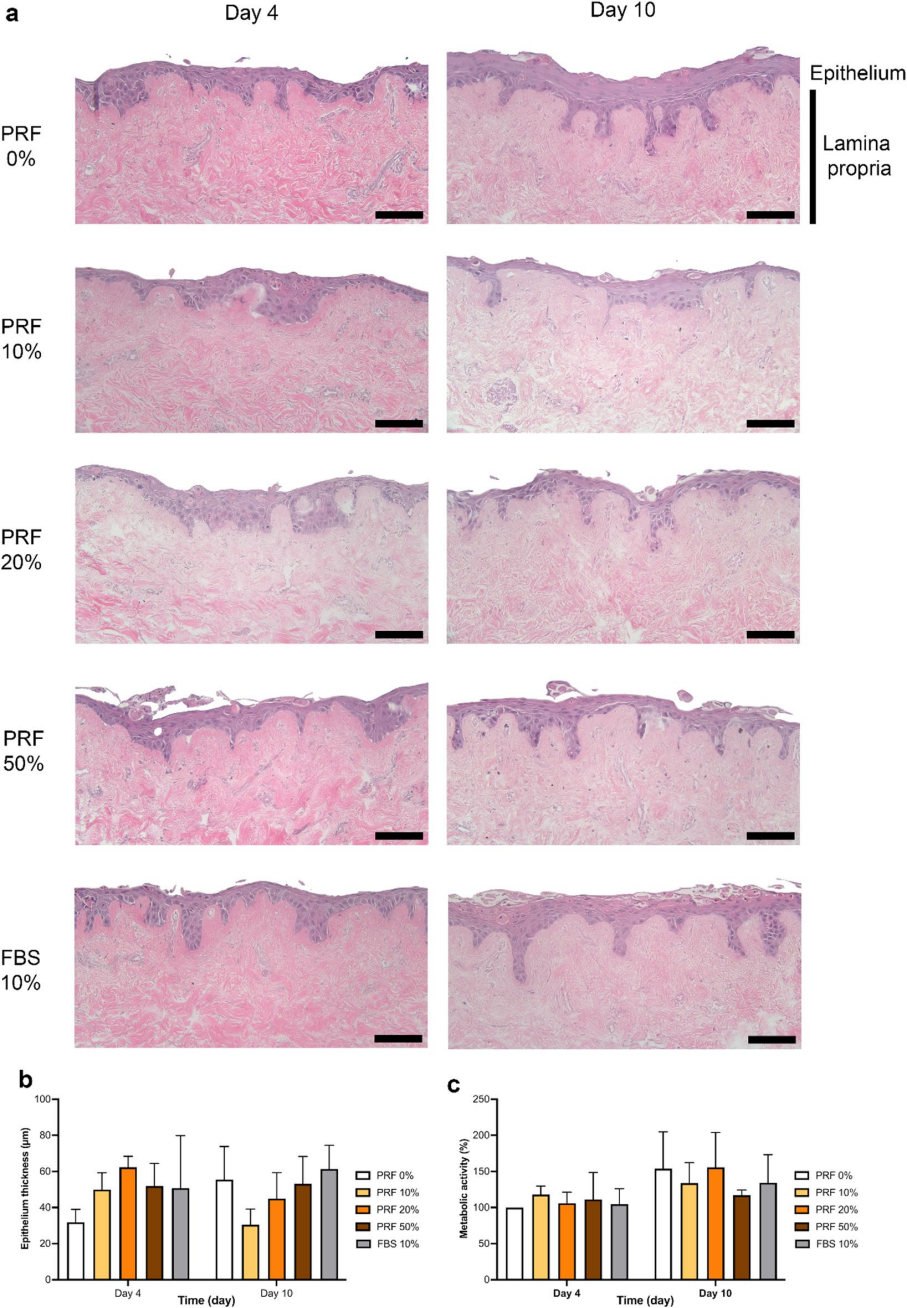
H&E-stained sections of tissue-engineered oral mucosa (TEOM) treated with conditioned medium derived from liquid-PRF after 4 and 10 days. The upper purple region of each image is the epithelium comprised of FN6/TERT cells, while the lower pink region is the fibroblast populated lamina propria. Representative images of TEOM sections cultured in conditioned medium containing PRF 0%, PRF 10%, PRF 20%, PRF 50% and FBS 10% after 4 and 10 days (**a**). The epithelium thickness (**b**) and the metabolic activity (**c**) of TEOM on day 4 and day 10 of culture (*N* = 3 *n* = 1). Mean ± standard deviation (S.D). Statistical analysis was performed using an ordinary one-way ANOVA followed by Dunnett’s multiple comparison against the control (PRF 0%) at each time point. Scale bar = 100 μm. Abbreviations; FBS, foetal bovine serum; PRF, platelet-rich fibrin.

To investigate the activity of liquid-PRF in a more physiologically relevant model, TEOM was cultured with liquid-PRF conditioned medium to assess its impact on epithelial development. The epithelial morphology of TEOM was evaluated following 4 and 10 days of conditioned media treatment (Fig. 4a). All TEOM had a multi-layered epithelium with densely packed cuboidal keratinocytes in the basal layer and one or two layers of flattened keratinocytes in the superficial layer. The morphology of TEOM following 4 or 10 days of treatment was comparable to those cultured with liquid-PRF conditioned media, the negative control (PRF 0%) and the positive control (FBS 10%).

The epithelium thickness was quantified and a slight increase in epithelial thickness was observed in all PRF-treated concentrations at day 4, followed by a decrease in epithelial thickness at day 10 in the PRF 10% condition, however, these differences were not statistically significant (Fig. 4b).

To assess the metabolic activity of TEOM, a resazurin reduction assay was performed (Fig. 4c). The results indicated that liquid-PRF had no significant effect on the metabolic activity of TEOM at any time point.

### Liquid-PRF showed no effect on keratinocyte proliferation in TEOM

**Fig. 5:**
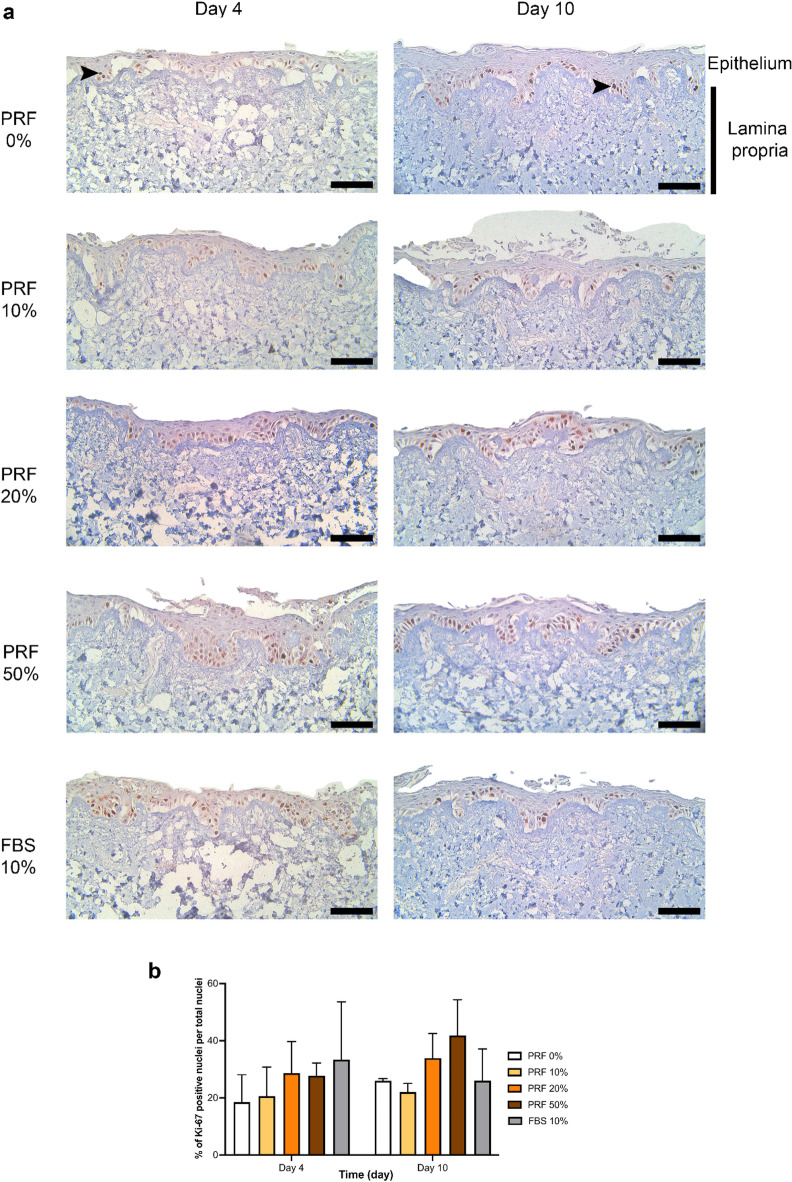
Immunohistochemistry staining for Ki-67 in tissue-engineered oral mucosa (TEOM) treated with conditioned medium derived from liquid-PRF after 4 and 10 days. The relative positions of the epithelium and lamina propria are labelled. Representative images of TEOM sections cultured in conditioned medium containing PRF 0%, PRF 10%, PRF 20%, PRF 50% and FBS 10% after 4 and 10 days (**a**). TEOM sections were stained with Ki-67 antibody (black arrows show examples of Ki67 positive, brown-stained nuclei). Quantification of Ki-67-positive nuclei (**b**) (*N* = 3, *n* = 2). Mean ± standard deviation (S.D). Statistical analysis was performed using an ordinary one-way ANOVA followed by Dunnett’s multiple comparison against the control (PRF 0%) at each time point. Scale bar = 100 μm. Abbreviations; FBS, foetal bovine serum; PRF, platelet-rich fibrin.

Following H&E staining, immunohistochemistry staining for Ki-67 was performed to identify proliferative cells within the epithelium. Figure 5a shows positive Ki-67 staining in the basal region of the epithelium of the TEOM for all experimental conditions. Examples of brown-stained, Ki-67 positive nuclei are indicated with arrows. 

On day 4, Ki-67-positive cells were observed in the suprabasal layer of the epithelium in TEOM treated with 50% PRF and 10% FBS (Fig. 5b), in contrast to the control group (0% PRF), where Ki-67-positive cells were restricted to the basal layer. A slight increase in the proportion of Ki-67-positive cells per total nuclei was detected in the PRF 20% and 50% models compared to the control (PRF 0%), however no significant differences were observed between any experimental conditions.

Following 10 days of treatment, Ki-67 positive cells were mostly localised in the basal layer of the epithelium. Again, an increase in Ki-67 positive cells was observed in TEOM treated with 20% and 50% liquid-PRF conditioned medium, however, this increase was not statistically significant.

## Discussion

There is great interest in the development of liquid-PRF therapies to promote oral regeneration as it is accessible, simple to prepare, autologous and biocompatible^5,6^. PRF has previously been shown to promote oral wound healing in some cases^5,19–21^; however, treatment outcomes remain inconsistent^27^. Existing clinical studies are mostly observational and the mechanistic function of liquid-PRF on oral soft tissue repair is not yet fully understood. The aim of this study was to investigate how factors secreted from liquid-PRF affected the behaviour of keratinocytes, fibroblasts and oral mucosa models in vitro.

Wound healing is a complex process regulated by a mixture of cytokines and growth factors, with temporal and spatial expression of different factors controlling regeneration^18^. Delayed healing in chronic wounds has been linked to an insufficient amount of growth factors such as PDGF, EGF, TGF-β, VEGF, and bFGF^18^. Since liquid-PRF comprises a mixture of bioactive factors at supraphysiological levels, we hypothesised that factors secreted from liquid-PRF could enhance the healing process by supplementing these bioactive factors.

Platelet counts confirmed the successful preparation of liquid-PRF using the horizontal-swing centrifugation method^20^. Conditioned media was collected over 72 h and the release of growth factors from the liquid-PRF was analysed to understand its biological properties. PDGF-BB, TGF-β1, and EGF were detected in the liquid-PRF conditioned medium, but not in basal DMEM. These growth factors are released during platelet degranulation^18^ with peak levels observed within 72 hours^5,28,29^ aligning with the incubation period used. Our findings are consistent with a previous study on other gel-like PRF formulations, where TGF-β1 and EGF were the most abundant growth factors in the conditioned medium^30^. Variability in the composition and function of liquid-PRF isolated from participants with different lifestyle and medical factors is well documented, however, the number of different donors used to generate liquid-PRF in this study (*n* = 9) was not sufficient to make comparisons between donor characteristics and liquid-PRF function.

Factors from liquid-PRF significantly increased the proliferation of oral fibroblasts in two-dimensional culture. This increase in fibroblast proliferation, driven by conditioned media from liquid-PRF, aligns with previous studies which showed an increase in fibroblast proliferation with liquid-PRF treatment compared to non-PRF conditions^5,19–21^. Our results also align with a case series showing that PRF membranes applied after anterior implant placement led to increased buccal mucosa thickness after three months, suggesting an increase in fibroblast proliferation and extracellular matrix formation (ECM)^33^. A study by Bi et al. further supports these findings, demonstrating that in vitro treatment of gingival fibroblasts with leukocyte- and platelet-rich fibrin led to the upregulation of genes involved in wound healing, including those regulating cell proliferation^36^.

The in vitro effects of liquid-PRF and other PRF derivatives on oral wound healing have been examined in oral fibroblasts, however little is known about its effects on oral keratinocytes^32^. In this study, factors secreted from liquid-PRF did not affect FNB6/TERT2 keratinocyte proliferation. EGF alone has been shown to increase keratinocyte proliferation^18^, however other growth factors found within liquid-PRF, such as TGF-β1, have been shown to inhibit the effects of EGF^34^, demonstrating the importance of studying liquid-PRF as a whole rather than individual growth factors in isolation.

The migration of keratinocytes and fibroblasts are essential for oral wound healing^31^ as cells migrate across the wounded area to facilitate wound closure and repair of the oral mucosa. A higher percentage of gap closure, representing an increase in cell migration, was observed when oral keratinocytes (FNB6/TERT) were treated with liquid-PRF conditioned media. EGF has previously been shown to promote keratinocyte migration during re-epithelialisation through the activation of the EGF receptor which is predominantly expressed in the basal layer of the epithelium^18^. Similarly, TGF-β1 has also been reported to promote keratinocyte migration by increasing the affinity of α3β1 integrin to ECM proteins^35^ and altering cellular polarity to a more migratory phenotype^34^.

In our study, the conditioned media derived from liquid-PRF stimulated keratinocyte migration; however, no significant effect was observed on fibroblast migration. A previous study demonstrated fibroblast treatment with PRF upregulated genes involved in migration^36^, however changes in gene expression and functional migration are distinct processes. Fibroblast migration in vivo is influenced by the extracellular matrix composition within the wound bed, therefore, the simplified, monolayer assay used here may not fully capture the complexity of the physiological wound environment. To address this limitation and to explore the effects of secreted factors in a more clinically relevant context, we employed a tissue-engineered oral mucosa model.

When TEOM models were treated with liquid-PRF conditioned media there was no significant effect on the metabolic activity, morphology, or epithelium thickness. Ki-67 staining, a marker for actively proliferating cells, revealed a slight increase in Ki-67 positive cells and more Ki-67 positive cells in the suprabasal layers of the TEOM treated with PRF conditioned media compared to untreated control models but these differences were not statistically significant. Together, this demonstrates there was no significant effect on epithelium development and cell proliferation following treatment with conditioned media from PRF in vitro.

Findings from our tissue engineered models differ from clinical studies reporting enhanced wound healing outcomes and improved soft tissue regeneration in periodontal surgeries and dental implant treatment following treatment with different formulations of PRF^13,39,40^. This discrepancy likely develops from fundamental differences between in vitro and in vivo environments and differences between the paracrine and direct effects of PRF formulations. Clinical healing involves complex interactions in functions including inflammation, immune response, vascularisation, and matrix remodelling which were not modelled here. Our in vitro TEOM models also lacked a defined wound edge, restricting the ability to directly assess the effects of liquid-PRF on the migration of keratinocytes across a wound, however the TEOM models were valuable to study cell proliferation and epithelial stratification, which are crucial for wound healing.

The FNB6/TERT cells used in this study produced a well-differentiated, stratified epithelium which was comparable to native oral epithelia and models generated using primary human oral keratinocytes^22^. Using a cell line provided greater consistency between models and we and others have previously demonstrated that FNB6/TERT cells behave comparably to primary oral keratinocytes^41^ and have cytokeratin and inflammatory gene expression profiles comparable to models generated from primary cells^22^. The epithelial morphology of models cultured in all the test conditions was comparable, including control TEOM (PRF 0%) which was cultured in basal media. This was unexpected, as we hypothesised that the lack of serum would restrict epithelial growth, however, our findings align with a previous study demonstrating that TEOM models can be constructed using serum-free culture medium^37^. The absence of serum components, along with the immortalized nature of the cell line used, may have increased the number of undifferentiated keratinocytes, enabling continued proliferation and formation of a multi-layered epithelium^37^.

Despite these limitations, the use of a full-thickness human 3D oral mucosa models presented here provided a reproducible platform to study the effects of liquid-PRF and its secreted factors on specific cell types in a way which is not possible in observational clinical studies and animal models. Future research is needed to better understand the mechanisms through which platelet concentrates, such as liquid-PRF, contribute to oral tissue repair. Incorporating wound models that simulate key features of clinical healing such as inflammation, vascularisation and wound edges will be essential to evaluating the regenerative potential of liquid-PRF in managing oral diseases and mucosal wounds in the future.

## Conclusion

In conclusion, this study demonstrates that liquid-PRF had no negative effects on tissue engineered oral mucosa and could enhance oral cell proliferation and migration in 2D culture. The presence of more Ki-67–positive cells in the suprabasal layers of TEOM cultured with liquid PRF conditioned media suggests a potential influence on epithelial stratification however further investigation is needed. The results from this study show that secreted factors from liquid-PRF were not able to increase epithelial thickness or oral mucosa metabolic activity in tissue engineered oral mucosa models. These results contrast some previous clinical studies which have demonstrated the positive effects of platelet concentrates on soft tissue regeneration, however supports other studies which show variable treatment outcomes and a lack of clinically significant improvements. Additional research on the impact of PRF in oral wound healing is necessary to gain deeper insight into its mechanisms of action, explore its potential in combination with other therapies, and strengthen the clinical evidence supporting its use. There is also a clear need for more well-designed, adequately powered, and controlled clinical studies.

## Data Availability

The data generated from this study are available upon reasonable request to the corresponding author.
